# The effectiveness of primary series CoronaVac vaccine in preventing COVID‐19 illness: A prospective cohort study among healthcare workers in Azerbaijan, May–November 2021

**DOI:** 10.1111/irv.13147

**Published:** 2023-10-03

**Authors:** Mark A. Katz, Madelyn Yiseth Rojas Castro, Nabil Seyidov, M. Trent Herdman, Samir Mehdiyev, C. Jason McKnight, Alina Guseinova, Radu Cojocaru, Jason Doran, Barbara Mühlemann, Christian Drosten, Javahir Suleymanova, Richard Pebody, Esther Kissling, Gahraman Hagverdiyev

**Affiliations:** ^1^ World Health Organization Regional Office for Europe Copenhagen Denmark; ^2^ Epiconcept Paris France; ^3^ Public Health and Reforms Center Ministry of Health Baku Azerbaijan; ^4^ UK Field Epidemiology Training Programme UK Health Security Agency London UK; ^5^ Institute of Virology, Charité–Universitätsmedizin Berlin, corporate member of Freie Universität Berlin Humboldt‐ Universität zu Berlin, and Berlin Institute of Health Berlin Germany; ^6^ German Centre for Infection Research (DZIF), partner site Charité Berlin Germany; ^7^ World Health Organization Country Office Baku Azerbaijan

**Keywords:** Azerbaijan, CoronaVac, COVID‐19, healthcare workers, vaccination, vaccine effectiveness

## Abstract

**Background:**

Healthcare workers (HCWs) have suffered considerable morbidity and mortality during the COVID‐19 pandemic. Few studies have evaluated the CoronaVac vaccine effectiveness (VE), particularly in Eastern Europe, where the vaccine has been widely used.

**Methods:**

We conducted a prospective cohort study among HCWs in seven hospitals in Baku, Azerbaijan between May 17 and November 30, 2021, to evaluate primary series (two‐dose) CoronaVac VE against symptomatic SARS‐CoV‐2 infection. Participants completed weekly symptom questionnaires, provided nasopharyngeal swabs for SARS‐CoV‐2 RT‐PCR testing when symptomatic, and provided serology samples at enrollment that were tested for anti‐spike and anti‐nucleocapsid antibodies. We estimated VE as (1 – hazard ratio)*100 using a Cox proportional hazards model with vaccination status as a time‐varying exposure, adjusting for hospital and previous SARS‐CoV‐2 infection status.

**Results:**

We enrolled 1582 HCWs. At enrollment, 1040 (66%) had received two doses of CoronaVac; 421 (27%) were unvaccinated. During the study period, 72 PCR‐positive SARS‐CoV‐2 infections occurred; 36/39 (92%) sequenced samples were classified as Delta variants. Primary series VE against COVID‐19 illness was 29% (95% CI: −51%; 67%) for the entire analysis period. For the Delta‐only period (July 1–November 30, 2021), primary series VE was 19% (95% CI: −81%; 64%). For the entire analysis period, primary series VE was 39% (95% CI: −40%; 73%) for HCWs vaccinated within 14–149 days and 19% (95% CI: −81%; 63%) for those vaccinated ≥150 days.

**Conclusions:**

During a period in Azerbaijan characterized by mostly Delta circulation, VE point estimates suggested that primary series CoronaVac protected nearly 1 in 3 HCWs against COVID‐19, but 95% confidence intervals were wide, with lower bounds that crossed zero, reflecting the limited precision of our VE estimates. Our findings underscore the need to consider booster doses for individuals who have received the primary series of CoronaVac.

## 1 INTRODUCTION

COVID‐19 vaccination has been shown to be a critical intervention to reduce morbidity and mortality from COVID‐19.^1^ Protecting healthcare workers (HCWs) through COVID‐19 immunization is essential for effective control of the COVID‐19 pandemic; HCWs are highly exposed to infection, have frequent contact with vulnerable patients, and are essential to the ongoing function of health services.^2^

Although many observational studies have evaluated the effectiveness of mRNA and viral vector vaccines, much less is known about the real‐world effectiveness of whole inactivated virus vaccines. Inactivated whole‐virus vaccines, which have the potential advantages of being easier to produce and store and also presenting a wider range of viral antigens to the immune system,^3^ accounted for nearly half of the 7.3 billion COVID‐19 vaccine doses delivered globally as of October 2021.^4^ Most of these doses have been used in low‐ and middle‐income countries (LMICs), where few studies have evaluated the effectiveness of COVID‐19 vaccines in general.^5^ In LMICs, differences in population demographics and differences in operational aspects of the vaccination campaign could potentially impact real‐world vaccine effectiveness (VE).^6^

CoronaVac (Sinovac, Beijing), a newly‐developed inactivated vaccine authorized for emergency use by the World Health Organization (WHO) in June 2021, has been widely used in countries in Central Asia and other countries in the eastern part of the WHO European Region^7^; however, only one study from the region—a study of HCWs in Turkey conducted during a period of Alpha variant predominance—has evaluated the effectiveness of this vaccine.^5^, ^7^

In Azerbaijan, an upper‐middle‐income country in the WHO European Region with a population of approximately 10 million people, the COVID‐19 vaccination campaign began on January 18, 2021. Populations considered most at risk, including HCWs, were prioritized for early vaccination. CoronaVac was the main vaccine available at the beginning of the vaccination campaign.

We conducted a prospective cohort study of COVID‐19 VE in HCWs in Azerbaijan. In this interim analysis of the study, we aimed to estimate the early VE of primary series (two‐dose) CoronaVac against PCR‐confirmed SARS‐CoV‐2 illness during May–November 2021.

## 2 MATERIALS AND METHODS

### 2.1 Study setting and population

The study design and analysis were guided by the WHO European Region HCW VE guidance document,^6^ and the study was conducted within the framework of WHO's Unity platform.^8^

From May 3 to July 17, 2021, we enrolled HCWs at seven hospitals in the Baku United Hospital network in Baku, the capital city of Azerbaijan, into a prospective cohort. We selected hospitals based on their accessibility to the study team in the context of quarantine‐related travel restrictions at the time. At the time of enrollment and during the study period, all sites admitted patients with COVID‐19.

We offered enrollment to all HCWs employed by participating hospitals for whom COVID‐19 vaccination was not contra‐indicated by previous allergies to vaccinations. National guidance at the time required HCWs to wait 6 months from a PCR‐confirmed infection before receiving the first dose. The national vaccination schedule recommended the administration of the second dose of CoronaVac 14–21 days after the first.^9^

Lists of all HCWs employed at study sites were provided by hospital directors to guide recruitment. We invited all HCWs to participate, including physicians, nurses, clinical support staff, and custodial workers, regardless of their COVID‐19 vaccination status, intention to get vaccinated in the future, or history of previous infection with SARS‐CoV‐2. At the time of enrollment, and throughout the analysis period, HCWs were required to have COVID‐19 vaccination in order to work in hospitals in Azerbaijan; however, because of the limited availability of the vaccine in much of the first half of 2021, this requirement was not strictly enforced.

### 2.2 Data collection and management

At enrollment, participants completed a questionnaire that included questions about demographics, comorbidities, previous SARS‐CoV‐2 infection, and SARS‐CoV‐2 vaccination history. Study staff then contacted participants weekly, using a standard questionnaire, to ask participants if they had experienced any symptoms in the past week (fever, cough, general weakness, fatigue, headache, muscle ache, sore throat, runny nose, shortness of breath, lack of appetite, nausea, vomiting, diarrhea, altered mental status, loss of taste, or loss of smell), and to ask about the details of any new COVID‐19 vaccines participants had received.

We advised all participants who became symptomatic to attend testing facilities at the study hospitals, where trained nurses collected nasopharyngeal swabs, which were tested for SARS‐CoV‐2 by RT‐PCR at on‐site government‐accredited laboratories or other Ministry of Health (MoH) laboratories. Symptomatic participants completed an additional survey that included information about the date of symptom onset, clinical care‐seeking, and details of PCR testing and results. PCR‐positive participants were interviewed again 30 days after their positive test, at which time further details about their course of illness, medical care, hospitalization, and complications were collected.

Data from interviewer‐led questionnaires and laboratory records were entered and stored securely using the Sorgular.az platform (Azerbaijan Public Health Reform Center). Participants' reports of positive PCR results were verified using the two national SARS‐CoV‐2 laboratory databases—the Etabib electronic medical records database^10^ and the MoH/Mandatory Health Insurance database—to which all SARS‐CoV‐2 PCR tests performed in public and private laboratories in the country are required to be reported. Participants' COVID‐19 vaccination history was verified using the national vaccine registry (Rendezvous, Azerbaijan MoH). Study staff contacted participants to resolve discrepancies between data from questionnaires and those from national databases, and to complete questions from study questionnaires that had not been answered.

### 2.3 Laboratory testing

Blood was collected from all participants at enrollment, and serum was stored at −20°C until testing. Sera samples were tested for anti‐spike antibodies with the Wantai SARS‐CoV‐2 total antibody ELISA (Beijing Wantai Biological Pharmacy, Beijing, China) and for anti‐nucleocapsid antibodies using the Platelia SARS‐CoV‐2 total antibody ELISA (Bio‐Rad Laboratories, Hercules, CA) in the InterDiagnostic Clinic Laboratory, Baku. The manufacturer's recommended controls and thresholds were used to define seropositivity. For participants who had hemolyzed or insufficient specimens, repeat blood draws were performed within 30 days of enrollment.

We selected a convenience sample for sequencing from PCR‐positive samples from participants at all study sites. SARS‐CoV‐2 PCR‐positive study samples were sent to the Charite University Institute of Virology laboratory in Berlin, Germany, for whole genome sequencing (WGS). Only samples with low cycle threshold values were sequenced.

### 2.4 Sample size estimation

We estimated that 1500 participants needed to be enrolled in the study to identify VE against symptomatic infection of 80% with an incidence of SARS‐CoV‐2 of 0.05 and vaccine coverage among participants of 80% to reach an 80% power level with alpha = 0.05. This estimate accounted for a likely drop‐out rate of roughly 10%.

### 2.5 VE analysis

For our primary study outcome, we measured two‐dose CoronaVac VE against symptomatic PCR‐confirmed COVID‐19 for the overall cohort. We considered a symptomatic COVID‐19 illness to be an event where the participant reported having symptoms anytime between 14 days before and 4 days after the swab date of the PCR‐positive test.

#### 2.5.1 Statistical model

VE was estimated as (1 – hazard ratio [HR])*100. HRs comparing vaccinated and unvaccinated individuals were estimated using Cox proportional hazards models with vaccination as a time‐varying exposure; the vaccination status of some individuals changed over time from unvaccinated to vaccinated, and therefore the same participant could contribute person‐time to both exposure categories. Study time was used as the underlying time scale in the Cox regression model. Participants were considered fully vaccinated 14 days after they received their second vaccine dose. Age was collected as years and successively grouped into five categories (20–29 years, 30–39 years, 40–49 years, 50–59 years, and 60+).

We calculated unadjusted and adjusted HRs and also calculated VE estimates. We categorized hospitals into two groups based on geographical location: three hospitals in central Baku were considered “central,” whereas four hospitals located on the outskirts of Baku were considered “peripheral.” Both unadjusted and adjusted HR estimates included hospital groups and prior infections as fixed effects. We assessed other prespecified potential confounders (e.g., month, age, sex, occupation, hands‐on care [which we defined as clinical HCWs who reported providing hands‐on medical care to patients], smoking, household size, any chronic condition, and BMI). In order to avoid overfitting the model, we included variables that changed the VE estimate by an absolute percentage of more than 5% using step‐wise backward selection.

We defined previous infection as a PCR‐confirmed infection prior to enrollment documented in either the Etabib database or the MoH/Mandatory Health Insurance database. Participants who were unvaccinated at enrollment and had PCR‐confirmed SARS‐CoV‐2 infection prior to enrollment began to contribute person‐time on the date they became eligible for vaccination (6 months after their last positive test). Participants who were vaccinated at enrollment and who had PCR‐confirmed COVID‐19 infection prior to enrollment were included in the analysis at the time point they were considered “at risk” of reinfection, which we defined as 90 days after their most recent positive PCR test.

Participants contributed person‐time from enrollment, or, for individuals with prior PCR‐confirmed SARS‐CoV‐2 infection, from the start of time at risk until the earliest outcome or exit from the study. Person‐time ended at whichever came first of the following outcomes: (1) the day of the first SARS‐CoV‐2 infection, designated by the date of symptom onset (for participants with symptomatic COVID‐19 infection) or by the swab date of the PCR test (for asymptomatic participants); (2) the day of receipt of a second COVID‐19 vaccination if it occurred before the recommended interval between the first and second dose; (3) the day of receipt of a third vaccine dose, or (4) the day of the last weekly questionnaire before complete loss to follow‐up, withdrawal from the study, transfer or retirement from their hospital of employment, death, or censor date for the analysis period (November 30, 2021). Participants were also censored from the primary analysis upon receiving a dose of any COVID‐19 vaccine other than CoronaVac. Because of the high level of completeness in the available data, we opted for a complete case approach.

#### 2.5.2 Further analyses and sensitivity analyses

We only conducted VE analyses for CoronaVac because few other vaccines were used in the study population. We evaluated VE during the overall study period (May 17–November 30, 2021) and separately for the period in which only SARS‐CoV‐2 B.1.617.2 viruses (the Delta variant) were sequenced (July 1, 2021–November 30, 2021), which we defined using WGS data from study samples along with publicly available data from Global Initiative on Sharing All Influenza Data (GISAID).^11^, ^12^ We also performed these two analyses excluding participants who had a PCR‐confirmed SARS‐CoV‐2 infection prior to enrollment. In addition, we examined VE since time since vaccination by comparing VE in the period from 14–149 days since the second vaccine dose to VE ≥ 150 days since the second vaccine dose.

We performed three sensitivity analyses. For the first, because reinfection can occur earlier than 90 days,^13^ we changed the definition of “time at risk” from 90 days after infection to 60 days after infection. For the second, because individuals may be protected as early as 7 days after their second dose,^14^ we defined “fully vaccinated” as 7 days after the second dose rather than 14 days after the second dose. Finally, we conducted a sensitivity analysis to evaluate the potential impact of unmeasured confounding using the E‐value approach, assuming both possible directions of bias—overestimation and underestimation.^15^

### 2.6 Ethical considerations

The study was approved by the WHO Research Ethics Review Committee (protocol: CERC.0097C) and the Ethics Committee of Azerbaijan State Academy of Physical Culture and Sport (March 3, 2021; Protocol #3/21). All participants provided informed, written consent. The study is registered in the clinicaltrails.gov registry (NCT050694).

## 3 RESULTS

We enrolled 1582 HCWs, which comprised 38.5% of eligible HCWs in participating hospitals. Three participants were excluded because no follow‐up data were obtained after enrollment (Figure S1).

Of the 1582 HCWs enrolled, 1473 (93%) were female; the median age was 49 (interquartile range [IQR]: 39–57); 408 (26%) were physicians; 591 (37%) were nurses; and 583 (37%) were other HCWs (Table 1). In all, 644 (41%) participants had at least one comorbidity, and 46 (3%) and 14 (1%) participants said they smoked currently or previously, respectively. A total of 458 (29%) HCWs had received the influenza vaccine in the 2020–2021 influenza season. Overall, 582 HCWs (37%) were from central hospitals in Baku, whereas 1000 HCWs (63%) were from peripheral Baku hospitals.

**TABLE 1:** Demographic, occupational, and clinical characteristics of participants at enrollment, by site, Azerbaijan, 2021.

Characteristic/category	Missing	All participants	Unvaccinated (any vaccine)	Partially vaccinated (1 dose CoronaVac)	Vaccinated (2doses CoronaVac)
Age	0	*n* = 1582	*n* = 421	*n* = 121	*n* = 1040
Median (IQR)		49 (39–57)	47 (38–57)	41 (37–54)	50 (40–58)
Age group	0	*n* = 1582	*n* = 421	*n* = 121	*n* = 1040
20–29, *n* (%)		84 (5)	27 (6)	14 (12)	43 (4)
30–39, *n* (%)		322 (20)	102 (24)	33 (27)	187 (18)
40–49, *n* (%)		426 (27)	104 (25)	37 (31)	285 (27)
50–59, *n* (%)		476 (30)	111 (26)	26 (21)	339 (33)
60+, *n* (%)		274 (17)	77 (18)	11 (9)	186 (18)
Sex	0	*n* = 1582	*n* = 421	*n* = 121	*n* = 1040
F, *n* (%)		1473 (93)	394 (94)	114 (94)	965 (93)
M, *n* (%)		109 (7)	27 (6)	7 (6)	75 (7)
Pregnant	1	*n* = 1472	*n* = 393	*n* = 114	*n* = 965
No, *n* (%)		1466 (100)	389 (99)	114 (100)	963 (100)
Yes, *n* (%)		6 (0)	4 (1)	0 (0)	2 (0)
Breastfeeding	1	*n* = 1472	*n* = 393	*n* = 114	*n* = 965
No, *n* (%)		1468 (100)	389 (99)	114 (100)	965 (100)
Yes, *n* (%)		4 (0)	4 (1)	0 (0)	0 (0)
					
Hospital	0	*n* = 1582	*n* = 421	*n* = 121	*n* = 1040
Hospital 15, *n* (%)		131 (8)	8 (2)	9 (7)	114 (11)
Hospital 18, *n* (%)		230 (15)	47 (11)	10 (8)	173 (17)
Hospital 23, *n* (%)		117 (7)	44 (10)	6 (5)	67 (6)
Hospital 24, *n* (%)		221 (14)	69 (16)	30 (25)	122 (12)
Hospital 26, *n* (%)		400 (25)	142 (34)	18 (15)	240 (23)
Hospital 29, *n* (%)		158 (10)	70 (17)	33 (27)	55 (5)
Hospital 7, *n* (%)		325 (21)	41 (10)	15 (12)	269 (26)
Hospital location	0	*n* = 1582	*n* = 421	*n* = 121	*n* = 1040
Central, *n* (%)		582 (37)	124 (29)	49 (40)	409 (39)
Periphery, *n* (%)		1000 (63)	297 (71)	72 (60)	631 (61)
Occupation/role in hospital	0	*n* = 1582	*n* = 421	*n* = 121	*n* = 1040
Other, *n* (%)		583 (37)	124 (29)	44 (36)	415 (40)
Nurse or midwife, *n* (%)		591 (37)	171 (41)	56 (46)	364 (35)
Medical doctor, *n* (%)		408 (26)	126 (30)	21 (17)	261 (25)
Hands‐on care*		*n* = 1582	*n* = 421	*n* = 121	*n* = 1040
Yes, *n* (%)		925 (58)	227 (54)	76 (63)	622 (60)
No, *n* (%)		657 (42)	194 (46)	45 (37)	418 (40)
Household size	0	*n* = 1582	*n* = 421	*n* = 121	*n* = 1040
1–3, *n* (%)		589 (37)	151 (36)	47 (39)	391 (38)
4–5, *n* (%)		747 (47)	194 (46)	56 (46)	497 (48)
6+, *n* (%)		246 (16)	76 (18)	18 (15)	152 (15)
Any chronic condition*	0	*n* = 1582	*n* = 421	*n* = 121	*n* = 1040
No, *n* (%)		938 (59)	220 (52)	78 (64)	640 (62)
Yes, *n* (%)		644 (41)	201 (48)	43 (36)	400 (38)
Smoking	0	*n* = 1582	*n* = 421	*n* = 121	*n* = 1040
Never smoked, *n* (%)		1522 (96)	407 (97)	116 (96)	999 (96)
Currently smokes, *n* (%)		46 (3)	11 (3)	4 (3)	31 (3)
Previously smoked, *n* (%)		14 (1)	3 (1)	1 (1)	10 (1)
Self‐assessed health status	0	*n* = 1582	*n* = 421	*n* = 121	*n* = 1040
Excellent, *n* (%)		106 (7)	20 (5)	7 (6)	79 (8)
Very good, *n* (%)		122 (8)	23 (5)	8 (7)	91 (9)
Good, *n* (%)		1046 (66)	268 (64)	73 (60)	705 (68)
Fair, *n* (%)		276 (17)	93 (22)	30 (25)	153 (15)
Poor, *n* (%)		32 (2)	17 (4)	3 (2)	12 (1)
Influenza vaccine 2020–2021	0	*n* = 1582	*n* = 421	*n* = 121	*n* = 1040
Yes, *n* (%)		458 (29)	85 (20)	22 (18)	351 (34)
No, *n* (%)		1124 (71)	336 (80)	99 (82)	689 (66)
Previous PCR‐confirmed COVID‐19 infection before enrollment	0	*n* = 1582	*n* = 421	*n* = 121	*n* = 1040
No, *n* (%)		1334 (84)	253 (60)	99 (82)	982 (94)
Yes, *n* (%)		248 (16)	168 (40)	22 (18)	58 (6)
Anti‐spike protein serology test results at enrollment	59	*n* = 1523	*n* = 406	*n* = 113	*n* = 1004
Positive, *n* (%)		1337 (88)	344 (85)	90 (80)	903 (90)
Negative, *n* (%)		186 (12)	62 (15)	23 (20)	101 (10)
Anti‐nucleocapsid protein serology test results at enrollment	65	*n* = 1517	*n* = 405	*n* = 113	*n* = 999
Positive, *n* (%)		1240 (82)	329 (81)	84 (74)	827 (83)
Negative, *n* (%)		261 (17)	70 (17)	27 (24)	164 (16)
Unknown/equivocal, *n* (%)		16 (1)	6 (1)	2 (2)	8 (1)
Seropositive at enrollment by either assay	0	*n* = 1582	*n* = 421	*n* = 121	*n* = 1040
Yes, *n* (%)		1423 (90)	363 (86)	97 (80)	963 (93)
No, *n* (%)		159 (10)	58 (14)	24 (20)	77 (7)
Time from receipt of second dose until start of person‐time contribution	542	*n* = 1040	*n* = 0	*n* = 0	*n* = 1040
Median (IQR)		99 (74–112) days			99 (74–112) days

* “Hands‐on care” refers to clinical HCWs who reported providing “hands‐on medical care to patients.”

** Chronic conditions include: cancer, chronic heart disease, high blood pressure/hypertension, chronic kidney disease, chronic liver disease (such as cirrhosis, hepatitis, and fatty liver disease), chronic lung disease (such as asthma and COPD), diabetes, immunocompromised (including solid organ transplant and HIV), neurologic disease (including cerebrovascular disease, epilepsy, and multiple sclerosis), obesity, and autoimmune disorder.

At enrollment, 1040 (66%) participants had received the primary series of CoronaVac, 121 (8%) participants had received only one dose of CoronaVac, and 421 (27%) were unvaccinated (Table 1). No participants had received other COVID‐19 vaccines at enrollment. Among those vaccinated with the primary series before enrollment, the median time since receipt of the second dose was 99 days (IQR: 74–112). Overall, 248 HCWs (16%) had a PCR‐positive COVID‐19 infection documented prior to enrollment, and 204/248 (84%) prior infections occurred before HCWs had received their first vaccine dose. Compared with unvaccinated participants, participants who had received the primary series vaccine before enrollment worked more commonly in central hospitals (39% vs. 29%), had more chronic conditions (38% vs. 48%) and had fewer PCR‐confirmed infections prior to enrollment (6% vs. 40%). Differences in participants by vaccination status at enrollment have been previously reported.^16^

In total, 963/1040 (93%) participants who had received the primary series vaccine were seropositive at enrollment for either anti‐nucleocapsid or anti‐spike protein antibodies; 903 (90%) were seropositive for anti‐spike antibodies, whereas 827 (83%) were seropositive for anti‐nucleocapsid antibodies. Among unvaccinated participants, 363/421 (86%) were seropositive to at least one of the two antibodies (Table 1). Of the 197 unvaccinated participants who did not report a previous PCR infection at enrollment, 136 (69%) were seropositive at enrollment by one of the two assays.

**FIGURE 1:**
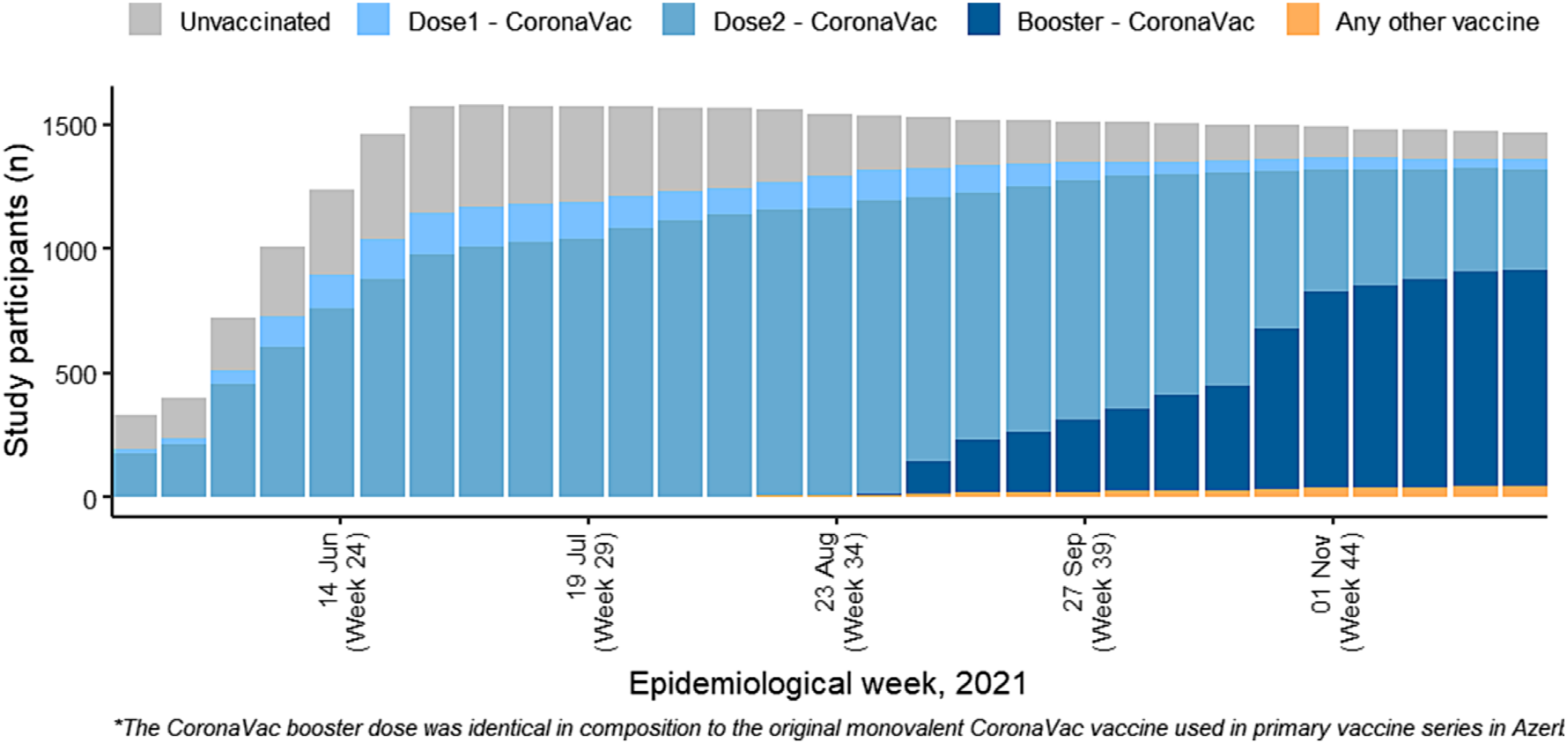
COVID‐19 vaccine coverage among study participants, by epidemiologic week, Azerbaijan, 2021.

During the follow‐up period, 10 (<1%) participants received primary series vaccination with vaccines other than CoronaVac (9 received Pfizer and 1 received Sputnik) and were excluded from the analysis. At their exit from analysis, 1485 (95%) participants had received the primary series of CoronaVac, 41 (3%) had received one dose of CoronaVac, and 43 (3%) remained unvaccinated. Changes in the vaccination status of the enrolled population over the course of the study are illustrated in Figure 1. At study exit, participants who had received the primary series were similar to unvaccinated participants in terms of median age, sex, smoking status, and self‐assessed health status; however, compared with participants who had received the primary series vaccine, unvaccinated participants were more commonly medical doctors (47% vs. 25%) and fewer unvaccinated participants received influenza vaccine during the previous season (70% vs. 81%) (Table S1).

The total follow‐up time in the study was 197,202 person‐days. Unvaccinated HCWs contributed a total of 24,745 person‐days to the 30‐week study period, while participants vaccinated with the primary series of CoronaVac contributed 172,457 person‐days. The median follow‐up time was 137 days (IQR = 114–156). During this time period, there were 380 symptomatic events among participants, of which 202 (53%) were tested for SARS‐CoV‐2 by PCR; 191/360 (53%) symptomatic events occurred among vaccinated participants, while 11/19 (57%) symptomatic events that occurred in unvaccinated participants were tested (*p* = 0.68).

**FIGURE 2:**
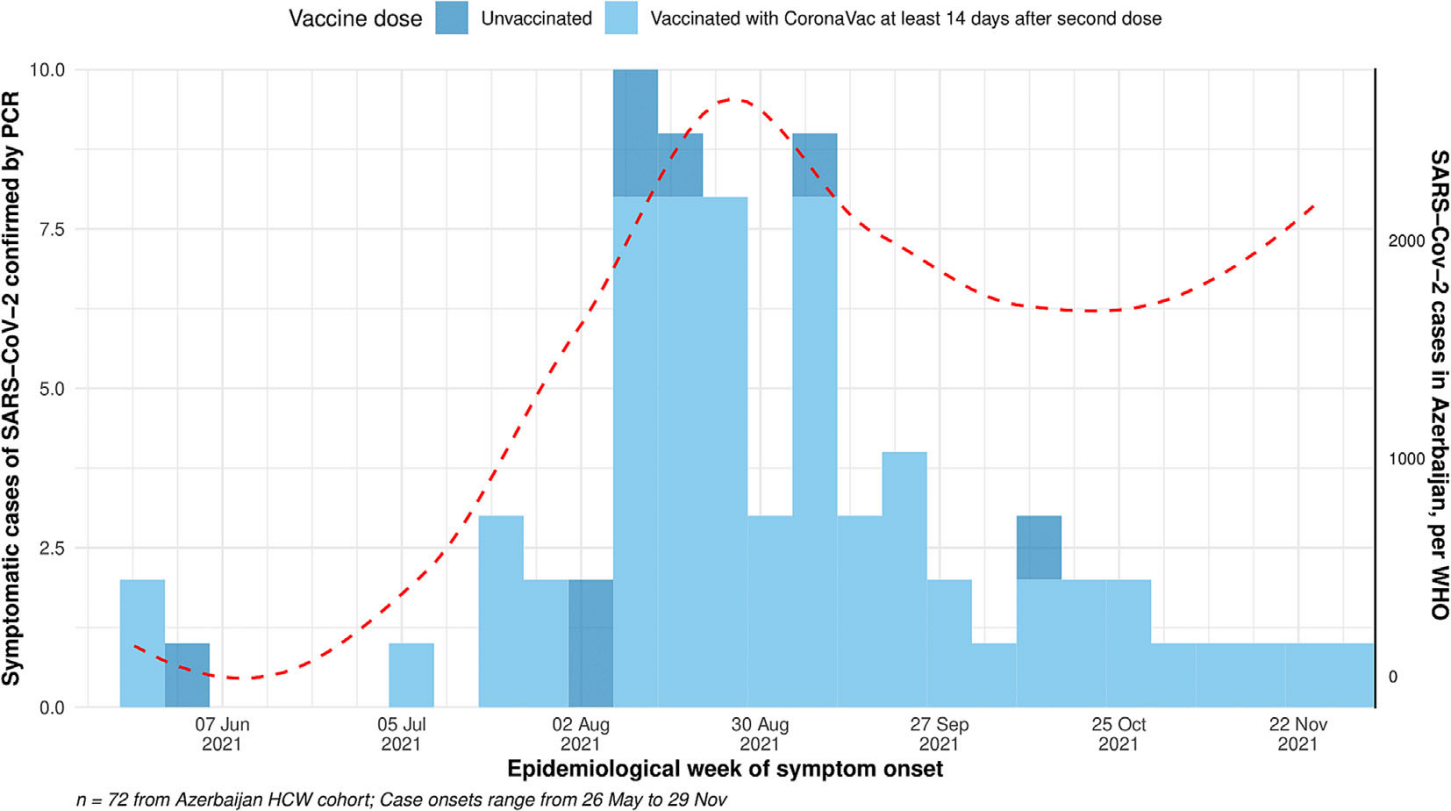
Epicurve showing COVID‐19 symptomatic cases by vaccination status in the study population (left *y*‐axis), and SARS‐CoV‐2 cases nationally (right *y*‐axis), by epidemiologic week, Azerbaijan, May 17–November 30, 2021. *Data on weekly SARS‐CoV‐2 incidence in Azerbaijan were taken from WHO COVID‐19 tracker.^28^

Overall, 72 participants (5%) had symptomatic PCR‐confirmed COVID‐19, including 64 vaccinated participants and 8 unvaccinated participants. Cases peaked in August, but incidence remained high through the end of the analysis period; the trajectory of cases in the study mostly mirrored the national trends of COVID‐19 incidence during the summer of 2021 in Azerbaijan (Figure 2). Of the 39 samples for which WGS data were available, 36/39 (92%) were Delta variants (Figure S2).

During the course of the analysis period, in the 30 days following their positive test, 44 participants with PCR‐confirmed COVID‐19 illness sought medical care (4 unvaccinated and 40 fully vaccinated), and 29 participants went to an emergency room (3 unvaccinated and 26 fully vaccinated). Three participants with PCR‐confirmed COVID‐19 illnesses were hospitalized (1 unvaccinated and 2 fully vaccinated). No deaths occurred among participants with PCR‐confirmed COVID‐19 illnesses. VE could not be calculated against these more severe outcomes because of the small number of events among unvaccinated participants.

**FIGURE 3:**
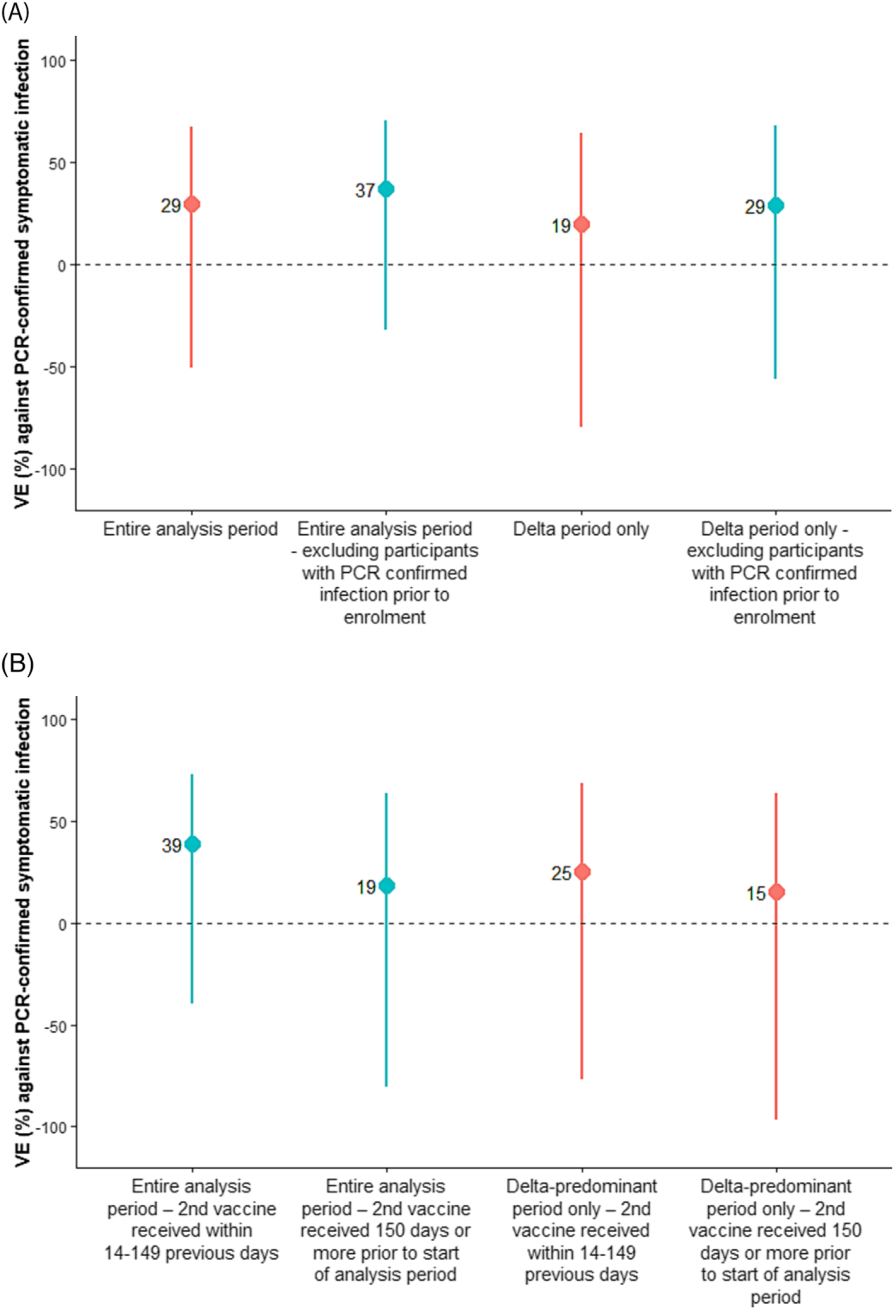
(A) Forest plot showing overall primary series CoronaVac vaccine effectiveness against PCR‐confirmed symptomatic SARS‐CoV‐2 illness for full cohort and for Delta‐predominant period only and excluding participants with PCR‐confirmed infection prior to enrollment (forest plots show point estimates and 95% CIs). (B) Forest plot showing overall primary series CoronaVac vaccine effectiveness against PCR‐confirmed symptomatic SARS‐CoV‐2 illness for entire cohort and for Delta‐predominant period only, by time from receipt of second vaccine (forest plots show point estimates and 95% CIs).

For the overall cohort, two‐dose VE was 29% (95% CI: −51%–67%) (Table 2, Figure 3A). For the Delta‐only period, two‐dose VE was 19% (95% CI: −81%–64%). VE was adjusted for previous infection only; no other potential confounders changed the VE estimates by more than 5% (Table S2). For the overall cohort analysis, vaccinated participants had received their second dose a median of 90 days (IQR: 75–112) prior to the beginning of the study period, while during the period of Delta circulation, vaccinated participants had received their second dose a median of 108 days (IQR: 106–132) prior to the analysis period.

**TABLE 2:** Two‐dose CoronaVac effectiveness against symptomatic PCR‐confirmed COVID‐19 infection for entire cohort and for entire cohort excluding participants who had a PCR‐confirmed SARS‐CoV‐2 infection prior to enrollment, during the total study period, and for Delta‐predominant period only, Azerbaijan, 2021.

	*N* participants	Total person‐time (days)	Symptomatic COVID‐19 infections	Unadjusted* HR	(95% CI)	Adjusted HR	(95% CI)	VE** (95% CI)
Total cohort*	1569							
Unvaccinated	415	24,745	8					
≥14 days from 2nd dose	1476	172,457	64	0.83	(0.40; 1.75)	0.71	(0.33; 1.51)	29.0 (−51.4; 66.7)
Total cohort, excluding participants with PCR‐confirmed infection prior to enrollment**	1327							
Unvaccinated	253	36,012	8					
≥14 days from 2nd dose	1268	304,360	61	0.63	(0.30; 1.33)	***	***	***
Delta period (July 1, 2021–November 30, 2021)								
Total cohort*	1565							
Unvaccinated	293	17,105	7					
≥14 days from 2nd dose	1478	147,488	62	0.94	(0.43; 2.1)	0.81	(0.36; 1.81)	19.1 (−80.7; 63.8)
Total cohort, excluding participants with PCR‐confirmed infection prior to enrollment**	1322							
Unvaccinated	181	21,634	7					
≥14 days from 2nd dose	1266	248,951	59	0.71	(0.34; 1.58)	***	***	***

*Note*: The total number of participants is not equal to the sum of the groups because one participant could have contributed person‐time to more than one group during the course of the analysis period.

Abbreviations: HR, hazard ratio; VE, vaccine effectiveness.

* Adjusted by hospital group.

** Adjusted by hospital group and previous infection status.

*** Adjusted VE could not be calculated.

When we excluded participants who had PCR‐confirmed SARS‐CoV‐2 infection prior to enrollment, the unadjusted HR was 0.63 (95% CI: 0.30–1.33), and the unadjusted HR during the Delta‐only period was 0.71 (95% CI: 0.34–1.58).

For the overall cohort, VE was 39% (95% CI: −40%–73%) for participants who had received their second CoronaVac vaccine within 14–149 days and 19% (95% CI: −81.5–63.4) for participants who had received their second CoronaVac vaccine ≥150 days prior (Table 3 and Figure 3B).

**TABLE 3:** Two‐dose CoronaVac effectiveness against symptomatic PCR‐confirmed COVID‐19 infection for entire cohort for total study period, and for Delta‐predominant period only, by time since receipt of second vaccine, Azerbaijan, 2021.

	*N* participants	Total person‐time (days)	Symptomatic COVID‐19 infections	Unadjusted HR (95% CI)	Adjusted HR (95% CI)	VE* (95% CI)
Total cohort	1569					
Unvaccinated [ref]	415	57,699	8			
14–149 days from 2nd dose	1467	196,338	24	0.68 (0.27; 1.53)	0.61 (0.27; 1.40)	38.6 (−40.3; 73.1)
≥150 days from 2nd dose	1069	143,107	40	1.01 (0.46; 2.22)	0.81 (0.37; 1.81)	18.5 (−81.5; 63.4)
Delta period (July 1, 2021–November 30, 2021)						
Total cohort	1565					
Unvaccinated [ref]	293	34,298	7			
14–149 days from 2nd dose	1467	163,194	22	0.81 (0.34; 1.91)	0.75 (0.32; 1.77)	25 (−77.5; 68.3)
≥150 days from 2nd dose	1072	117,861	40	1.04 (1.54; 2.39)	0.85 (0.37; 1.98)	14.9 (−97.7; 63.4)

*Note*: The total number of participants is not equal to the sum of the groups because one participant can contribute to the person‐time simultaneously in several groups.

Abbreviations: HR, hazard ratio; VE, vaccine effectiveness.

* Adjusted by hospital group and previous infection status.

In sensitivity analyses, when we decreased the period after infection that participants were considered not at risk from 90 to 60 days, results were very similar (<1% difference) to the 90‐day analysis (Table 4). When we considered a participant to be fully vaccinated at 7 days instead of 14 days, VE differed by <2% from the results of the primary analysis.

**TABLE 4:** Two‐dose CoronaVac effectiveness against symptomatic PCR‐confirmed COVID‐19 infection for full cohort for entire study period (a) when re‐infection could occur 60 days (rather than 90 days) after infection; and (b) when participants were considered fully vaccinated 7 days (rather than 14 days) after their second dose.

	*N* participants	Total person‐time (days)	Symptomatic COVID‐19 infections	Incidence per 10,000 person‐days	Unadjusted HR (95% CI)	Adjusted HR (95% CI)	VE* (95% CI)
a. Reinfection definition: 60 days instead of 90 days							
Total cohort	1569						
Unvaccinated	415	57,699	8	1			
≥14 days from 2nd dose	1476	340,967	64	2	0.83 (0.39; 1.75)	0.71 (0.33; 1.51)	29.2 (−50.7; 66.7)
							
b. Fully vaccinated with primary series vaccine defined at 7 days after vaccination, instead of 14 days							
Total cohort	1569						
Unvaccinated	415	62,551	8	1			
≥14 days from 2nd dose	1481	347,010	64	2	0.82 (0.39; 1.72)	0.70 (0.33; 1.48)	30.4 (−48.1; 67.3)

*Note*: The total number of participants is not equal to the sum of the groups because one participant can contribute to the person‐time simultaneously in several groups.

Abbreviations: HR, hazard ratio; VE, vaccine effectiveness.

* Adjusted by hospital group and previous infection status.

Our calculation of E‐values indicated that an unmeasured confounder would have had to have had at least associations of ≥3 with both the exposure and the outcome to fully explain away our observed VE of the primary analysis if the true VE was outside of 0%–50% (Figure S3).

## 4 DISCUSSION

We found that during a period in Azerbaijan characterized by mostly Delta circulation, our VE point estimate suggested that the primary series of CoronaVac protected nearly 1/3 of HCWs against COVID‐19. However, our wide 95% CIs, with lower bounds that crossed zero (−51%–67%), reflect the limited precision of our estimates. Our findings are the first to describe COVID‐19 VE in the South Caucasus region—an area of the WHO European Region that continues to have much lower rates of vaccination compared with western and central Europe.^17^ As of November 2022, less than half of the adult population (48%) in Azerbaijan had completed the primary series vaccination for COVID‐19, and less than 10% had completed a booster dose.^7^

Our primary course CoronaVac VE point estimates of 37% for the entire analysis period and 29% for the Delta‐only period (equivalent to unadjusted HRs of 0.63 and 0.71, respectively) when we excluded participants who had previous PCR‐confirmed infection are similar to previous studies of CoronaVac VE against symptomatic infection and all infections. A study of HCWs in Turkey without previous infection, the only published study of CoronaVac VE in the WHO European Region, found that adjusted two‐dose VE against SARS‐CoV‐2 infection was 39% (HR 0.61, 95% CI: 0.46–0.80) during a period of Alpha variant circulation.^7^ That same study reported an unadjusted two‐dose CoronaVac VE of 48% (95% CI: 29%–61%) against symptomatic infection. Studies outside of the WHO European Region that have evaluated two‐dose CoronaVac VE during periods of predominant Delta circulation showed a range of results that varied in part according to the time since vaccination. A study from Brazil that included individuals who were 60–179 days and >180 days after their second dose found two‐dose VE against symptomatic disease of 37.6% (95% CI: 36.1–39.1) and 34% (95% CI: 32.3–35.7), respectively, in a Delta‐predominant period.^18^ In contrast, two studies from Asia found higher two‐dose VE against illness and infection during periods of Delta‐predominant circulation, but these studies included individuals who had mostly been recently vaccinated. A study from Thailand that used the test‐negative design reported two‐dose CoronaVac effectiveness of 60% (95% CI: 49–69) against infection; vaccinated participants in that study had received their last dose a mean of 81 days (range: 60–91 days) prior to the analysis period.^19^ A study related to an outbreak of the SARS‐CoV‐2 Delta variant in China reported a two‐dose CoronaVac VE against illness rate of 73.0 (95% CI: 22.3–90.6) among individuals who had mostly received their second vaccine dose within the previous 3 months.^20^

For individuals who received the primary vaccine series, VE against infection during Delta‐predominant periods has been shown to be mostly higher for mRNA and viral vector vaccines compared with CoronaVac.^21^ However, against Omicron, two‐dose VE has been much lower against symptomatic infection and, to a lesser extent, severe disease across vaccine products.^22^ Homologous and heterologous monovalent booster doses have been shown to increase protection against both mild and severe COVID‐19 illnesses in Delta and Omicron.^22^ In Azerbaijan, booster doses were recommended for HCWs in September 2021. Both CoronaVac and Comirnaty (Pfizer/Biontech) have been offered without a preferential recommendation. To date, few studies have evaluated the VE of a heterologous booster compared with a homologous booster following primary series vaccination with CoronaVac. In Brazil, during an Omicron‐predominant period, for individuals who had received the primary series of CoronaVac, the heterologous booster with Comirnaty had a higher VE than the homologous booster with CoronaVac against both symptomatic infections (56.8% [95% CI: 56.3–57.3] vs. −2.9% [95% CI: −5.2–0.6]) and severe disease (86% [95% CI: 84.5–87.4) vs. 73.6% [95% CI: 63.9–80.7]) for those vaccinated 8–59 days prior to the analysis period.^18^ Differences persisted for those boosted >59 days prior. However, in Hong Kong Special Administrative Region, also during a period of Omicron BA.2 circulation, VE against mortality and severe complications was mostly similar for individuals who had received a primary series of CoronaVac followed by Comirnaty as a booster compared with those who had received three doses of CoronaVac.^23^ Both studies showed increased VE against all endpoints for booster doses compared with the primary series. In Azerbaijan, where less than 10% of the adult population has received a booster vaccine,^24^ conveying the important benefits of booster doses to the public is critical.

Our study population likely had high rates of previous infection at enrollment. Because vaccination with inactivated vaccine leads to seroconversion in both anti‐spike and anti‐nucleocapsid antibody tests, we could not use antibody testing to determine previous infection among participants who had been vaccinated prior to enrollment. However, 69% of unvaccinated HCWs who did not report a previous infection were seropositive by at least one of the two antibody tests at enrollment, and these findings likely reflect the extent of previous infection in the overall study population. Despite more than two of every three participants likely having been previously infected, we still found some benefit, albeit not statistically significant, to primary series vaccination with CoronaVac. The added benefit of primary COVID‐19 vaccination and booster doses in previously infected individuals (hybrid immunity) has been widely demonstrated in other studies.^25^

In our study, we found a trend towards decreased VE among participants for whom more than 5 months had passed since their second CoronaVac vaccine. Waning VE with increased time since COVID‐19 vaccination has been described for CoronaVac and other COVID‐19 vaccines.^26^, ^27^ The waning effectiveness of primary series vaccination again underscores the importance of booster doses to increase protection.

Our study has a number of strengths. Because we enrolled and systematically followed a discrete cohort of HCWs, we were able to obtain data about vaccination status, SARS‐CoV‐2 test results, and clinical outcomes, information that would not have been discernible from routinely collected data. The protocol was followed rigorously; participants completed more than 95% of the weekly symptom questionnaires during the study period. Only 34 (2%) participants were lost to follow‐up. Finally, the use of serology at enrollment allowed us to estimate the prevalence of prior infections among unvaccinated participants and also provided information on seroconversion rates among HCWs who received the inactivated CoronaVac vaccine.

Our study also has some limitations. First, because of the relatively low number of events, the relatively low amount of person‐time among unvaccinated participants, and the relatively low VE, our VE estimates had wide confidence intervals. The imprecision of the point estimates in our study limited our ability to draw firm conclusions regarding VE. The study was not powered to estimate VE against severe outcomes like hospitalization and death—critical endpoints for vaccine evaluation. The study may have suffered from selection bias because enrollment was voluntary; however, it is not obvious in which way this bias would have impacted the magnitude and direction of the VE estimates. We did not have access to demographic and clinical data for the entire HCW population of the study hospitals or the country for comparison. However, we were able to compare participants who remained in the study with those who were lost to follow‐up; regarding the major confounder in our analysis, previous infection, we found only small differences (12% among those lost to follow‐up vs. 16% among participants who remained in the study).

In our study, although there were no differences in age and sex between unvaccinated and vaccinated participants, there were more unvaccinated physicians at the end of the study period, and a slightly lower percentage of vaccinated participants had chronic diseases compared with unvaccinated participants. This latter finding may have led to a “healthy vaccinee effect,” resulting in an overestimation of the VE. However, as we mention in the results section, self‐reported health status was not a confounder in our study. In addition, a few participants remained unvaccinated at the end of the study, and these unvaccinated participants may differ from vaccinated participants in ways that we did not measure, including the likelihood of exposure to SARS‐CoV‐2 and other parameters. Although we do not know in which direction and to what extent unmeasured confounding could have impacted our VE estimates, sensitivity analyses indicate that it is likely that any unmeasured confounding would have masked a true VE of 0%–50%. Otherwise, the associations of the unmeasured confounder would have had to have had an HR of at least ≥3 with the outcome and the exposure, which we deem unlikely.

More unvaccinated participants (40%) than vaccinated participants (6%) had a PCR‐confirmed SARS‐CoV‐2 infection prior to enrollment—likely the result of the Azerbaijan MoH recommendation to defer vaccinating individuals who had been infected with SARS‐CoV‐2. Although differential rates of previous infection in the two arms could bias VE estimates, we accounted for this difference by including only participants eligible for vaccination, according to the recommendations, and including previous SARS‐CoV‐2 infection as a confounding variable. Finally, only 53% of symptomatic events were tested for SARS‐CoV‐2; however, the difference in the percentage of symptomatic events that were tested in vaccinated participants compared with unvaccinated participants was small and not statistically significant.

In conclusion, during a period in Azerbaijan characterized by mostly Delta circulation, VE point estimates suggested that primary series CoronaVac protected nearly 1 in 3 HCWs against COVID‐19, but 95% CIs were wide, with lower bounds that crossed zero (−51%–67%), reflecting the limited precision of our VE estimates. HCWs in our cohort had high rates of previous infection and had mostly received their second vaccine three months previously. These findings reaffirm previous findings about the limited durability of protection of the primary series of CoronaVac and other vaccines in preventing symptomatic infection and thus should provide further support for the consideration of booster doses. Our findings also support the utility of COVID‐19 vaccination even among individuals who have been previously infected, a policy that is currently promoted in Azerbaijan and elsewhere and that will be critical to continue as more of the population in Azerbaijan and globally have experienced at least one COVID‐19 infection. As this is an ongoing cohort study, future analyses will evaluate VE in the context of booster doses and Omicron infection in Azerbaijan.

## AUTHOR CONTRIBUTIONS

Gahraman Hagverdiyev, Mark A. Katz, Nabil Seyidov, Samir Mehdiyev, and Richard Pebody conceived the cohort study on which this analysis is based. Mark A. Katz, Nabil Seyidov, M. Trent Herdman, Samir Mehdiyev, C. Jason McKnight, Alina Guseinova, Radu Cojocaru, Jason Doran, Javahir Suleymanova, Richard Pebody, Gahraman Hagverdiyev, Madelyn Yiseth Rojas Castro, and Esther Kissling planned and implemented the study, including the development of study protocols, data quality checks, and acquisition of data. Mark A. Katz, M. Trent Herdman, Nabil Seyidov, Gahraman Hagverdiyev, and Samir Mehdiyev conceived the article. Mark A. Katz and M. Trent Herdman drafted the manuscript and performed the literature search. Madelyn Yiseth Rojas Castro performed the data analysis with support from Esther Kissling, Samir Mehdiyev, Nabil Seyidov, and Gahraman Hagverdiyev. Madelyn Yiseth Rojas Castro and Esther Kissling directly accessed and verified the raw data and take responsibility for the integrity and accuracy of the analyses. Barbara Mühlemann and Christian Drosten performed sequencing of study samples. All authors contributed to the interpretation of the results and critically revised the manuscript. All authors had full access to all the data reported in the study and accept responsibility to submit the paper for publication.

## CONFLICT OF INTEREST STATEMENT

The authors report there are no competing interests to declare.

### PEER REVIEW

The peer review history for this article is available at https://www.webofscience.com/api/gateway/wos/peer-review/10.1111/irv.13147.

## Supporting information


**Figure S1.** Flowchart illustrating the enrollment of healthcare workers in COVID‐19 vaccine effectiveness study, Azerbaijan, 2021

**Figure S2.** Whole genome sequencing results of SARS‐CoV‐2 positive cases from the vaccine effectiveness study (N = 39) and from GISAID data for Azerbaijan (N = 2) by week during the study period, May 17 – November 30, 2021 (N = 41).*

**Figure S3.** E‐value quantification to assess potential unmeasured confounding in the association between CoronaVac primary series vaccination and COVID‐19 illness in a cohort study of healthcare workers in Azerbaijan, 2021.
*For whole genome sequencing of PCR‐positive study samples, total nucleic acids were extracted using the Roche MagNAPure 96. Library preparation was performed using the NimaGen EasySeq™ SARS‐CoV‐2 WG Seq kit (NimaGen, Nijmegen, The Netherlands) according to manufacturer's instructions. DNA libraries were sequenced on an Illumina MiSeq machine (300 cycles, paired‐end). Reads were aligned to the SARS‐CoV‐2 reference sequence (GISAID accession EPI_ISL_402125) using bowtie2 (version 2.4.4). The consensus was called using ivar (version 1.3.1) Lineages were assigned using pangolin version 4.0.6. Sequences are available on GISAID under accession numbers EPI_ISL_15714246‐EPI_ISL_15714285.

**Table S1.** Participant demographics and clinical characteristics by COVID‐19 vaccination status on the last day of follow‐up,* Azerbaijan, 2021
**Table S2.** Fully adjusted CoronaVac Vaccine effectiveness against symptomatic COVID‐19 infection for full cohort.

## Data Availability

The data are not publicly available due to privacy or ethical restrictions. The authors do not have consent from study participants to share the data.
