# Supplementary figures and images for: The effectiveness of primary series CoronaVac vaccine in preventing COVID‐19 illness: A prospective cohort study among healthcare workers in Azerbaijan, May–November 2021
Source: Influenza Other Respir Viruses. 2023 Oct 3;17(10):e13147. doi: 10.1111/irv.13147 (PMC10548024; doi:10.1111/irv.13147)

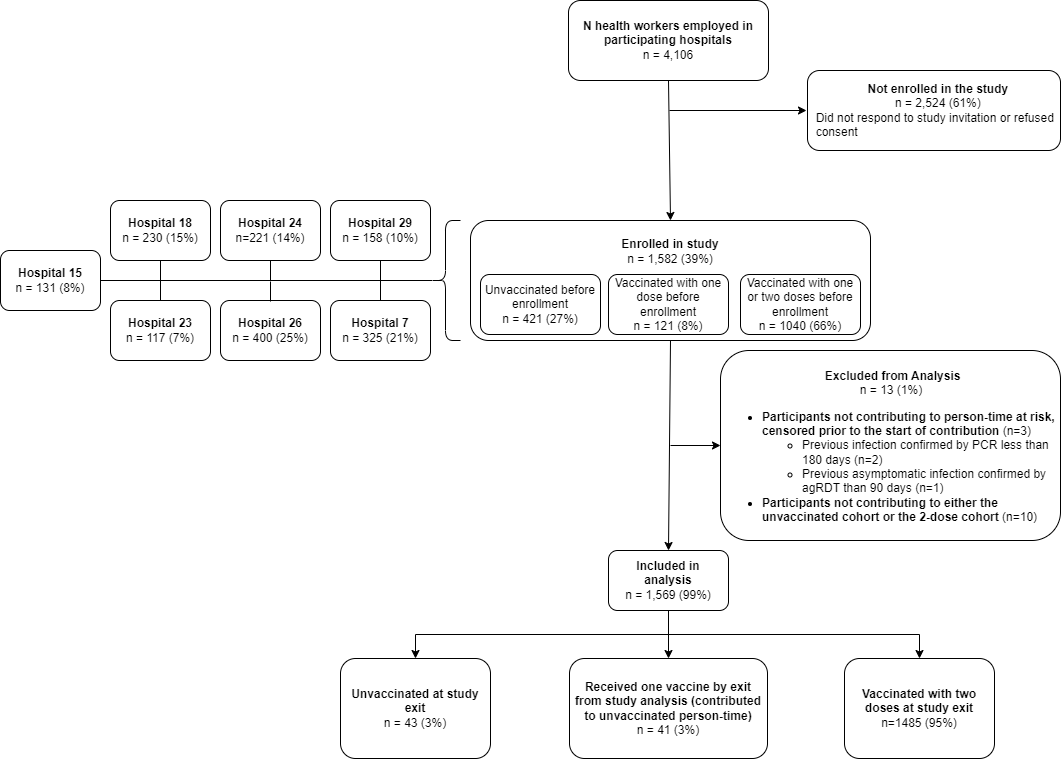

Supplement: Supplementary file 1 — Figure S1. Flowchart illustrating the enrollment of healthcare workers in COVID‐19 vaccine effectiveness study, Azerbaijan, 2021 [file IRV-17-e13147-s004.png]

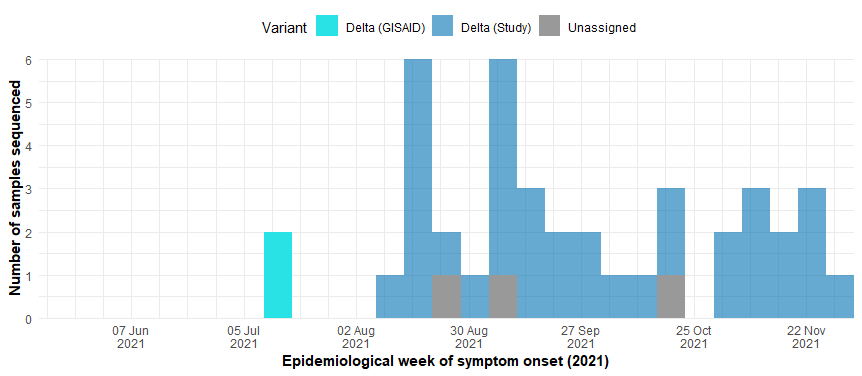

Supplement: Supplementary file 2 — Figure S2. Whole genome sequencing results of SARS‐CoV‐2 positive cases from the vaccine effectiveness study (N = 39) and from GISAID data for Azerbaijan (N = 2) by week during the study period, May 17 – November 30, 2021 (N = 41).* [file IRV-17-e13147-s003.png]

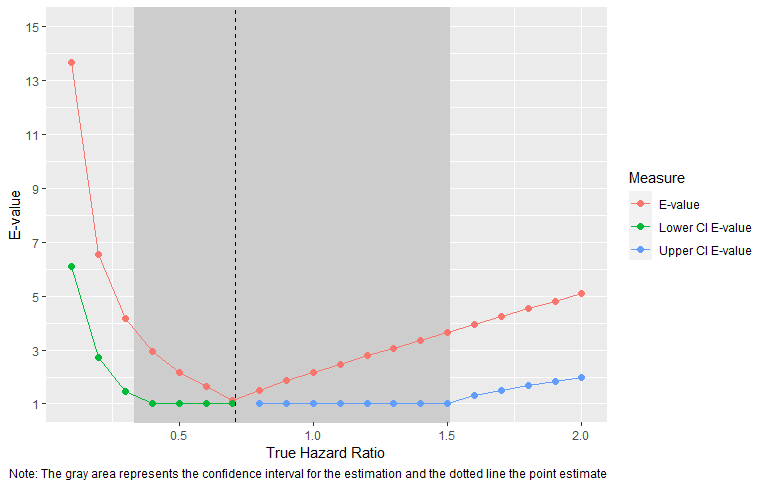

Supplement: Supplementary file 3 — Figure S3. E‐value quantification to assess potential unmeasured confounding in the association between CoronaVac primary series vaccination and COVID‐19 illness in a cohort study of healthcare workers in Azerbaijan, 2021. *For whole genome sequencing of PCR‐positive study samples, total nucleic acids were extracted using the Roche MagNAPure 96. Library preparation was performed using the NimaGen EasySeq™ SARS‐CoV‐2 WG Seq kit (NimaGen, Nijmegen, The Netherlands) according to manufacturer's instructions. DNA libraries were sequenced on an Illumina MiSeq machine (300 cycles, paired‐end). Reads were aligned to the SARS‐CoV‐2 reference sequence (GISAID accession EPI_ISL_402125) using bowtie2 (version 2.4.4). The consensus was called using ivar (version 1.3.1) Lineages were assigned using pangolin version 4.0.6. Sequences are available on GISAID under accession numbers EPI_ISL_15714246‐EPI_ISL_15714285. [file IRV-17-e13147-s001.png]
