# Supplementary material for: The effectiveness of primary series CoronaVac vaccine in preventing COVID‐19 illness: A prospective cohort study among healthcare workers in Azerbaijan, May–November 2021
Source: Influenza Other Respir Viruses. 2023 Oct 3;17(10):e13147. doi: 10.1111/irv.13147 (PMC10548024; doi:10.1111/irv.13147)
Supplement: Supplementary file 4 — Table S1. Participant demographics and clinical characteristics by COVID‐19 vaccination status on the last day of follow‐up,* Azerbaijan, 2021 Table S2. Fully adjusted CoronaVac Vaccine effectiveness against symptomatic COVID‐19 infection for full cohort. [file IRV-17-e13147-s002.docx]

**SUPPLEMENTARY TABLES**

**Supplementary Table 1.** Participant demographics and clinical characteristics by COVID-19 vaccination status on the last day of follow-up,* Azerbaijan, 2021

| **Characteristic/Category** | **Missing** | **All Participants** | **Unvaccinated** | **Partially vaccinated (1 dose CoronaVac)** | **Vaccinated with primary series (two doses CoronaVac)** |
| --- | --- | --- | --- | --- | --- |
| **Age** | 0 | n= 1569 | n= 43 | n= 41 | n= 1485 |
| Median (IQR) |  | 49 (39-57) | 51 (36.5-61.5) | 42 (38-57) | 49 (40-57) |
| **Age group** | 0 | n= 1569 | n= 43 | n= 41 | n= 1485 |
| 20-29, n(%) |  | 84 (5) | 5 (12) | 1 (2) | 78 (5) |
| 30-39, n(%) |  | 319 (20) | 11 (26) | 15 (37) | 293 (20) |
| 40-49, n(%) |  | 423 (27) | 4 (9) | 8 (20) | 411 (28) |
| 50-59, n(%) |  | 471 (30) | 8 (19) | 11 (27) | 452 (30) |
| 60+, n(%) |  | 272 (17) | 15 (35) | 6 (15) | 251 (17) |
| **Sex** | 0 | n= 1569 | n= 43 | n= 41 | n= 1485 |
| F, n(%) |  | 1460 (93) | 41 (95) | 38 (93) | 1381 (93) |
| M, n(%) |  | 109 (7) | 2 (5) | 3 (7) | 104 (7) |
| **Pregnant** | 1 | n= 1459 | n= 41 | n= 38 | n= 1380 |
| No, n(%) |  | 1453 (100) | 38 (93) | 38 (100) | 1377 (100) |
| Yes, n(%) |  | 6 (0) | 3 (7) | 0 (0) | 3 (0) |
| **Breastfeeding** | 1 | n= 1459 | n= 41 | n= 38 | n= 1380 |
| No, n(%) |  | 1455 (100) | 39 (95) | 38 (100) | 1378 (100) |
| Yes, n(%) |  | 4 (0) | 2 (5) | 0 (0) | 2 (0) |
|  |  |  |  |  |  |
| **Hospital** | 0 | n= 1569 | n= 43 | n= 41 | n= 1485 |
| Hospital 15, n(%) |  | 131 (8) | 0 (0) | 1 (2) | 130 (9) |
| Hospital18, n(%) |  | 227 (14) | 7 (16) | 4 (10) | 216 (15) |
| Hospital 23, n(%) |  | 114 (7) | 5 (12) | 7 (17) | 102 (7) |
| Hospital 24, n(%) |  | 219 (14) | 10 (23) | 6 (15) | 203 (14) |
| Hospital 26, n(%) |  | 397 (25) | 6 (14) | 19 (46) | 372 (25) |
| Hospital 29, n(%) |  | 157 (10) | 11 (26) | 3 (7) | 143 (10) |
| Hospital 7, n(%) |  | 324 (21) | 4 (9) | 1 (2) | 319 (21) |
| **Hospital Location** | 0 | n= 1569 | n= 43 | n= 41 | n= 1485 |
| Central, n(%) |  | 577 (37) | 17 (40) | 11 (27) | 549 (37) |
| Periphery, n(%) |  | 992 (63) | 26 (60) | 30 (73) | 936 (63) |
| **Occupation/Role in hospital** | 0 | n= 1569 | n= 43 | n= 41 | n= 1485 |
| Other, n(%) |  | 578 (37) | 11 (26) | 13 (32) | 554 (37) |
| Nurse or Midwife, n(%) |  | 586 (37) | 12 (28) | 19 (46) | 555 (37) |
| Medical Doctor, n(%) |  | 405 (26) | 20 (47) | 9 (22) | 376 (25) |
| **Hands-on care**** |  | n= 1569 | n= 43 | n= 41 | n= 1485 |
| **Yes, n(%)** |  | 652 (42) | 22 (51) | 17 (41) | 613 (41) |
| **No, n(%)** |  | 917 (58) | 21 (49) | 24 (59) | 872 (59) |
| **Household size** | 0 | n= 1569 | n= 43 | n= 41 | n= 1485 |
| 1-3, n(%) |  | 584 (37) | 20 (47) | 15 (37) | 549 (37) |
| 4-5, n(%) |  | 742 (47) | 14 (33) | 19 (46) | 709 (48) |
| 6+, n(%) |  | 243 (15) | 9 (21) | 7 (17) | 227 (15) |
| **Any chronic condition***** | 0 | n= 1569 | n= 43 | n= 41 | n= 1485 |
| No, n(%) |  | 933 (59) | 27 (63) | 23 (56) | 883 (59) |
| Yes, n(%) |  | 636 (41) | 16 (37) | 18 (44) | 602 (41) |
| **Smoking** | 0 | n= 1569 | n= 43 | n= 41 | n= 1485 |
| Never smokes, n(%) |  | 1510 (96) | 41 (95) | 40 (98) | 1429 (96) |
| Currently smokes, n(%) |  | 46 (3) | 2 (5) | 0 (0) | 44 (3) |
| Previously smokes, n(%) |  | 13 (1) | 0 (0) | 1 (2) | 12 (1) |
| **Self-assessed health status** | 0 | n= 1569 | n= 43 | n= 41 | n= 1485 |
| Excellent, n(%) |  | 105 (7) | 2 (5) | 5 (12) | 98 (7) |
| Very good, n(%) |  | 121 (8) | 2 (5) | 4 (10) | 115 (8) |
| Good, n(%) |  | 1039 (66) | 28 (65) | 24 (59) | 987 (66) |
| Fair, n(%) |  | 274 (17) | 9 (21) | 6 (15) | 259 (17) |
| Poor, n(%) |  | 30 (2) | 2 (5) | 2 (5) | 26 (2) |
| **Received influenza vaccine during 2020-2021** | 0 | n= 1569 | n= 43 | n= 41 | n= 1485 |
| Yes, n(%) |  | 454 (29) | 8 (19) | 5 (12) | 441 (30) |
| No, n(%) |  | 1115 (71) | 35 (81) | 36 (88) | 1044 (70) |

*refers to the end of the person-time included in the analysis for each participant. The person-time ended on: 1) the day of the first infection, 2) the day of receipt of a third vaccine dose, 3) the day of receipt of a second vaccine dose if the interval between first and second dose is shorter than the manufacturer recommendation, 4) the day of the last weekly questionnaire before complete loss to follow-up, withdrawal or censor date, or the last weekly questionnaire before 1 Dec 2021.

** “Hands-on care” refers to clinical HCWs who reported providing “hands-on medical care to patients”

***chronic conditions include: cancer, chronic heart disease, high blood pressure/hypertension, chronic kidney disease, chronic liver disease (such as cirrhosis, hepatitis, fatty liver disease), chronic lung disease (such as asthma, COPD), diabetes, immunocompromised (including solid organ transplant and HIV), neurologic disease (including cerebrovascular disease, epilepsy, multiple sclerosis), obesity, autoimmune disorder

**Supplementary Table 2.** Fully adjusted CoronaVac Vaccine effectiveness against symptomatic COVID-19 infection for full cohort.

|  | **N participants** | **Total person-time (days)** | **Symptomatic COVID-19 infections** | **Adjusted HR** | **(95%CI)** | **Adjusted VE*** | **(95%CI)** |
| --- | --- | --- | --- | --- | --- | --- | --- |
| **Total Cohort** | 1569 | 197142 |  |  |  |  |  |
| Unvaccinated | 415 | 24725 | 8 |  |  |  |  |
| ≥14d from 2nd dose | 1475 | 172417 | 64 | 0.71 | (0.33 - 1.52) | 29.1 | (-51.7; 66.9) |

*Hospital Location, PCR previous infection, age group, sex, any chronic condition, health status, smoking, hospital job, household size

Hospital location was divided into Central (hospitals located in central Baku) and Periphery (Hospitals located in the periphery of Baku)
